# Comparison of endovascular therapy care and outcome in primary and comprehensive stroke centers for acute ischemic stroke in China a real-world nationwide registry

**DOI:** 10.3389/fneur.2025.1655954

**Published:** 2025-11-10

**Authors:** Ting-yu Yi, Shujuan Gan, Meihua Wu, Weifeng Huang, Yanmin Wu, Hanming Tu, Changjun Yang, Lihua Xu, Hui Tan, Lin Gao, Shuguang Zhao, Linping Wei, Yingchun Wu, Guiguan Yang, Jian Ye, Dongsheng Ju, Ya Shao, Zaihui Zhang, Junfeng Su, Shijun Zhao, Weidong Huang, Xinshan Wu, Dinglai Lin, Xiaohui Lin, ZhiNan Pan, Xiufen Zheng, Ganji Hong, Rongcheng Chen, Lisan Zeng, Thanh N. Nguyen, Xuesong Bai, Liqun Jiao, Wen-huo Chen

**Affiliations:** 1 Cerebrovascular and Neuro-Intervention Department, Zhangzhou Affiliated Hospital of Fujian Medical University, Fujian, China; 2 Interventional Neuroradiology, Department of Neurology, Beijing Tiantan Hospital, Capital Medical University, Beijing, China; 3 Department of Neurology, The Second Hospital of Zhangzhou, Fujian, China; 4 Department of Cerebrovascular Disease, Fujian Medical University Union Hospital, Fuzhou, China; 5 Department of Neurology, Yongkang First People’s Hospital, Zhejiang, China; 6 Department of Neurology, Sinan County People’s Hospital of Guizhou province, Zhejiang, China; 7 Department of Neurology, Jiamusi Central Hospital, Heilongjiang, China; 8 Department of Neurology, Taoyuan Country People’s Hospital of Hunan province, Hunan, China; 9 Department of Neurology, Fukang General Hospital of Liaoning Health Industry Group, Liaoning, China; 10 Department of Neurology,Taihe County People’s Hospital, Anhui, China; 11 Department of Neurology, Longchuan County People’s Hospital, Guangdong, China; 12 Department of Neurology,ORDOS central hospital, Neimenggu, China; 13 Department of Neurology, Xingguo People’s Hospital, Jiangxi, China; 14 Department of Neurology, Fuqing Hospital Affiliated of Fujian Medical University, Fujian, China; 15 Department of Neurology, Songyuan Jilin Oilfield Hospital, Songyuan, Jilin, China; 16 Department of Cerebrovascular Disease, Gansu Provincial Hospital of TCM, Gansu, China; 17 Department of Neurology, Xiu Yan People’s Hospital, Liaoning, China; 18 Department of Neurology, Jingzhou Hospital Affiliated to Yangtze University, Hubei, China; 19 Department of Interventional, Tangshanfengrun county People’s Hospital, Shandong, China; 20 Department of Neurology, Xinhui People’s Hospital, Guangdong, China; 21 Department of Neurology, Bayinggolin Mongolian Autonomous Prefecture People’s Hospital, Xinjiang, China; 22 Department of Neurology, Boston University School of Medicine, Boston Medical Center, Boston, MA, United States; 23 Department of Neurosurgery, Xuanwu Hospital, Capital Medical University, Beijing, China; 24 Department of Neurosurgery, China International Neuroscience Institute (China-INI), Beijing, China; 25 Department of Interventional Neuroradiology, Xuanwu Hospital, Capital Medical University, Beijing, China

**Keywords:** endovascular treatment, prognosis, large vessel occlusion, acute ischemia, primary stroke center, comprehensive stroke center

## Abstract

**Background and purpose:**

Endovascular treatment (EVT) is a standard therapy for acute ischemic stroke (AIS) caused by large vessel occlusion (LVO). However, the performance of EVT in primary stroke centers (PSCs) in China remains uncertain. This study aims to explore the performance of EVT in PSCs and compare it with that in comprehensive stroke centers (CSCs).

**Methods:**

We conducted a prospective registry of EVT at 11 CSCs and 26 PSCs in China. AIS patients with intracranial LVO who received EVT were divided into two groups based on the type of stroke center. We compared the AIS workflow, EVT procedural details, radiological, and clinical outcomes between the two groups.

**Results:**

From November 2021 to December 2022, 1,196 patients were enrolled, and 847 were included in the analysis. Overall, 84.8% of patients achieved successful reperfusion, and 46.3% achieved good clinical outcomes. Compared with patients treated at CSCs, those treated at PSCs had shorter onset-to-presentation time (OPT: 152 min vs. 268 min, *p* < 0.001) but longer door-to-puncture time (DPT: 112 min vs. 95 min, *p* < 0.001). Successful reperfusion rates were lower in PSCs (76.6% vs. 91.0%, *p* < 0.001), and mortality was higher (24.7% vs. 14.4%, p < 0.001). However, good clinical outcomes were similar between the two groups (44.3% vs. 47.8%, *p* = 0.309).

**Conclusion:**

In China, successful reperfusion rates were lower and mortality rates were higher despite shorter onset-to-presentation times in primary stroke centers. Additionally, door-to-puncture times were prolonged despite limited use of advanced brain imaging. These findings highlight the need for EVT skill training and improvement in the AIS workflow in primary stroke centers to enhance patient outcomes.

## Introduction

Endovascular therapy (EVT) is the standard treatment for acute ischemic stroke caused by large vessel occlusion (AIS-LVO) (1). The efficacy of EVT is influenced by multiple factors, including the medical capabilities of hospitals and their staff, such as the use of EVT devices, the EVT skills of neuro-interventionalists, and the establishment of emergency treatment green channels for AIS. Additionally, other factors such as traffic conditions, public education, and awareness of AIS also play important roles, which may vary significantly between developed and developing countries. The RESILIENT randomized trial in Brazil demonstrated that patients with AIS-LVO in a developing country can still benefit from EVT (2).

Differences in stroke care are evident not only between developed and developing countries but also between county and municipal-level hospitals. Given China’s vast geographical expanse, there is significant variation in the construction of stroke centers, stroke burden, and the quality of stroke care across different regions (3). To improve the quality of stroke care, the guidelines for stroke center construction in China, issued in 2015, recommended establishing two levels of stroke centers: primary stroke centers (PSCs), typically county-level hospitals, and comprehensive stroke centers (CSCs), usually municipal or provincial hospitals (4). The efficacy of reperfusion treatments, including intravenous thrombolysis (IVT) and endovascular thrombectomy (EVT) for AIS is time depend (5). Therefore, reperfusion therapy should be performed as soon as possible. The “2019 Stroke Report in China” showed that EVT was increasingly performed in PSCs. However, differences in medical care capabilities, emergency treatment green channels for AIS, and traffic conditions may exist between PSCs and CSCs. We hypothesize that the AIS workflow and medical care capabilities of hospitals and their staff are suboptimal in PSCs. To explore these differences, we conducted a prospective real-world registry study, named the “Endovascular Treatment for Acute Ischemic Stroke in Chinese Municipal and County Hospitals (the ETERNITY registry),” from November 2021 to December 2022. We hope the findings will provide valuable information for improving stroke care quality in China.

## Methods

### Hospitals and participants

The ETERNITY registry is a nationwide, prospective, observational study of consecutive adult patients with AIS patients due to LVO who underwent EVT. The study was conducted across 11 CSCs(municipal hospital) and 26 PSCs(county hospitals) certified by the Ministry of Health China Stroke Prevention Project Committee (CSPPC) (6). CSCs serve as the backbone of the regional stroke care system. These centers, also known as advanced stroke centers, are tertiary hospitals responsible for stroke diagnosis, treatment, education, training, and scientific research. They are subject to national unified quality control management and actively promote the implementation of key stroke prevention recommendations. CSCs also facilitate the establishment of a two-way referral mechanism within their region to enhance overall stroke care (4). PSCs also referred to as Stroke Prevention Centers, adhere to standards that include establishing a green channel for multi-disciplinary collaboration in emergency management, participating in regional stroke classification treatment networks, conducting stroke prevention and secondary prevention programs, and ensuring endovascular thrombectomy capability (4).

This study included hospitals from 19 provinces and 1 municipality across China. The study protocol was approved by the Ethics Committees of local hospital and all participating centers. Written informed consent was obtained from patients or their legally authorized representatives. The study is registered at https://www.clinicaltrials.gov with the unique identifier number.

Patients were enrolled according to the following inclusion criteria: (1) age ≥18 years; (2) diagnosis of AIS based on imaging-confirmed intracranial LVO, including isolated cervical internal carotid artery occlusion or tandem occlusion, or occlusion in the intracranial internal carotid artery (ICA), middle cerebral artery (MCA, M1/M2), or anterior cerebral artery [ACA, A1/A2, 7]; (3) National Institutes of Health Stroke Scale (NIHSS) score ≥ 6; (4) Alberta Stroke Program Early CT Score (ASPECTS) ≥ 6;(5). initiation of any type of EVT, including mechanical thrombectomy or emergent angioplasty via balloon and stent; (6) onset-to-puncture time within 24 h, and if onset-to-presentation time (OPT) was within 6–24 h, computed tomography perfusion (CTP) should be performed and the results of CTP should fulfil the DAWN (7) or DEFUSE (8) criteria. The exclusion criteria were (1) no follow-up outcome data; (2) posterior circulation stroke and (3) no evidence of an LVO on angiogram.

### Data collection

The baseline information included patients’ demographic data, medical history, physical examination findings, imaging results, and time-metric data. Treatment details included the administration and timing of IVT and EVT procedure information. Before enrollment, all sites were uniformly trained on an electronic data capture system, which allowed for data entry and electronic signatures. All the electronic data were captured with central quality checks by blinded statisticians to control for consistency, plausibility and completeness.

### Clinical outcome assessment

The follow-up clinical examinations included NIHSS score assessments of the patients’ neurological function and modified Rankin Scales (mRS) assessments of the outcomes at 90 days, and a good outcome defined as a mRS score ≤ 2. The 90 days follow-up was ascertained using a standardized telephone interview performed by trained investigators blinded to the baseline and procedural data. The stroke subtypes were determined according to the Trial of ORG 10172 in Acute Stroke Treatment (TOAST) classifications (9). Symptomatic intracranial hemorrhage (sICH) was defined as an increase of ≥ 4 points in the NIHSS scores according to the Heidelberg criteria (10).

### Radiological assessment

Baseline imaging assessments, including non-contrast CT, magnetic resonance (MR) imaging, CT angiography (CTA), MR angiography (MRA), and digital subtraction angiography (DSA), as well as post-procedural CT results, were evaluated by an imaging core laboratory using a standardized protocol (DISCOM). All analyses were performed by personnel blinded to clinical data and outcomes. All imaging assessments were independently conducted by two experienced neuroradiologists (each with over 5 years of experience), with a third neuroradiologist available for adjudication when discrepancies arose. The radiological evaluations encompassed the following: ASPECTS evaluated on plain CT for anterior circulation stroke, location of the occlusion site, presence of underlying ICAS (11, 12), baseline and postprocedural extended thrombolysis in cerebral infarction (eTICI) (13), tandem occlusion, and occurrence of ICH on post-treatment imaging. Successful reperfusion was defined as an eTICI ≥ 2b. Procedural-related complications such as vessel dissection, vasospasm, vessel perforation, embolization into new territory (ENT) and distal embolism(DE) (14) were also recorded. For patients whose images could not be obtained, site-reported data were used. The imaging review criteria of the local investigators were the same as those of the core laboratory.

### Statistical analysis

Continuous data are presented as medians and interquartile ranges (IQRs) or means and standard deviations (SDs); between-group differences were tested by *t* tests and Mann–Whitney *U* tests, respectively. Categorical variables are presented as proportions and were compared using the *χ*^2^ test. Patients were classified into CSCs and PSCs group, baseline characteristic, radiological and clinical outcome were compared between two group. Typical variables related with prognosis (age, NIHSS, ASPEC, OPT, occlusion site) combined with stroke center type were included in multivariate logistic regression analyses. Odds ratios (ORs) were computed to determine the associated relationships between clinical outcome and stroke center type. All analyses were performed using SPSS (version 15.0; SPSS, Inc., Chicago, IL, United States), with a significance level of *p* < 0.05 (two-sided).

## Results

### Participated hospital and included patient

**Figure 1:**
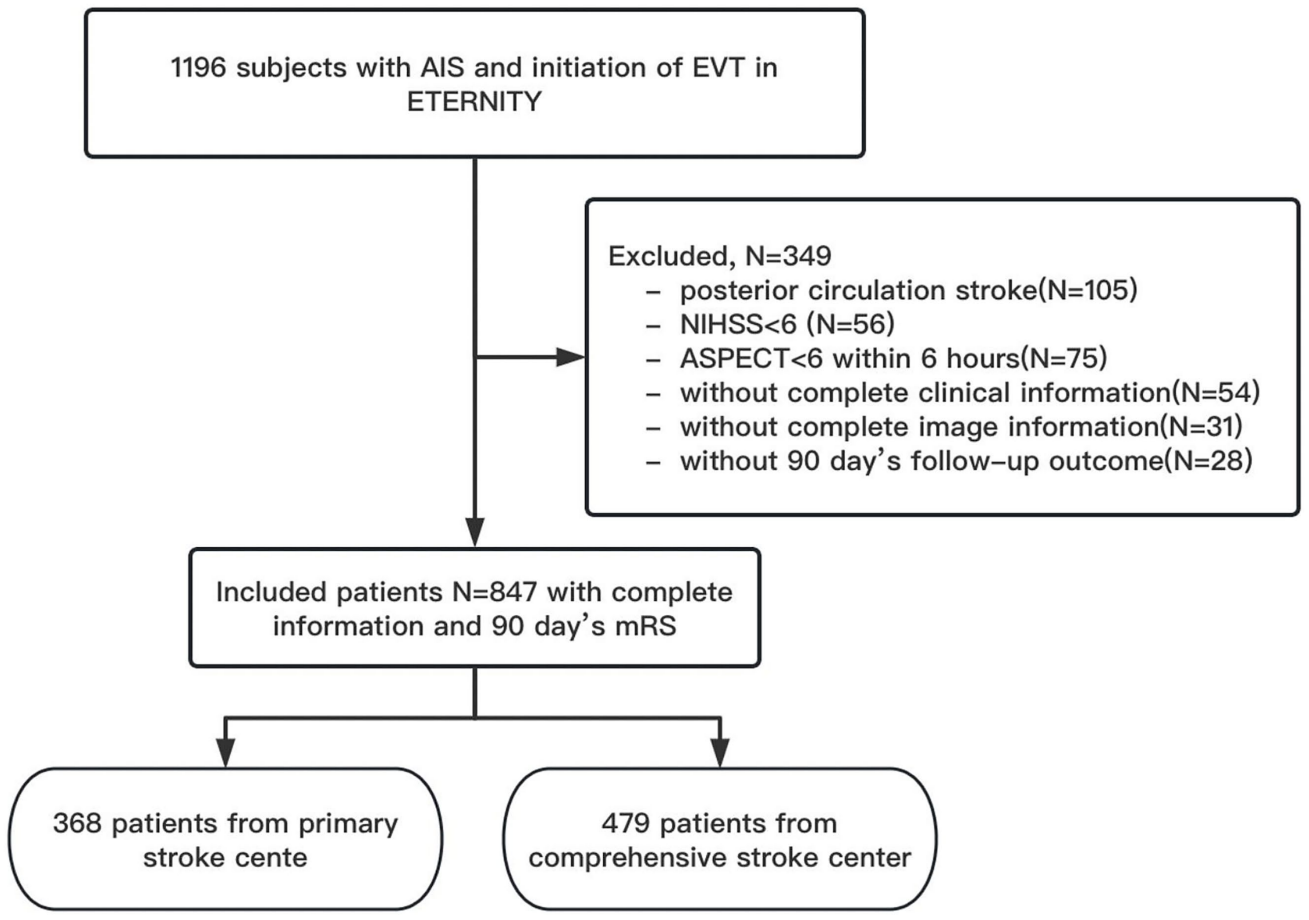
Study flow chart. NIHSS indicated National Institutes of Health Stroke Scale; ASPECT, Alberta Stroke Program Early CT Score.

Between November 2021 and December 2022, totally 11 CSCs and 26 PSCs from 19 provinces and 1 municipality in China participated the study. In total, 1,196 subjects with AIS were registered (Supplementary Table 1). Of these, 349 were excluded: (1) 105 with posterior circulation stroke; (2) 56 with NIHSS scores < 6; (3) 75 with ASPECT < 6; (4) 54 without complete clinical information; (5) 31 who lacked complete imaging information and (6) 28 without 90-day follow-up data. Ultimately, this study included 847 patients, comprising 368 from PSCs and 479 from CSCs, respectively (Figure 1).

### Baseline characteristic of included patients

The baseline characteristic of included 847 patients were showed in Tables 1, 2, the mean age was 68 ± 12 years, 62%(525/847) were men, the median admission NIHSS scores was 14. Regarding vascular risk factors, 49.4% (418/847) had hypertension, 20.4% (173/847) had diabetes, 16.5% (33/847) had hyperlipidemia, 34.7% (294/847) had atrial fibrillation (AF), and 28.9% (245/847) were smokers. In terms of imaging modality, all patients underwent non-contrast CT, with a median ASPECTS of 8. Additionally, 66.1% (560/847) of patients underwent angiography imaging, and 37.0% (313/847) underwent perfusion imaging. According to the TOAST classification, 33.4% (283/847) had large-artery atherosclerosis (LAA), 48.2% (408/847) had cardioembolic (CE), and 18.4% (156/847) had other undefined strokes. Regarding occlusion site, 38.5% (326/847) were in the ICA, 58.6% (496/847) in the MCA, and 2.9% (25/847) in the ACA; 13.8% (117/847) showed tandem occlusion.

**Table 1:** Demographics and characteristics of the included patients, stratified by stroke center type.

Parameters	All (*n* = 847)	PSCs (*n* = 368)	CSCs (*n* = 479)	*p* value
Age (mean ± SD)	68 ± 12	68 ± 12	67 ± 12	0.408
Male sex (*n*, %)	525 (62.0%)	230 (62.5%)	295 (61.6%)	0.786
Risk factors (*n*, %)				
Hypertension	418 (49.4%)	183 (49.7%)	235 (49.1%)	0.847
Diabetes	173 (20.4%)	73 (19.8%)	100 (20.9%)	0.710
Hyperlipidemia	33 (16.5%)	10 (8.9%)	23 (26.1%)	0.001
Atrial fibrillation	294 (34.7%)	151 (41.0%)	143 (29.9%)	< 0.001
Smoking	245 (28.9%)	122 (33.2%)	123 (25.7%)	0.017
TOAST classification (*n*, %)				
LAA	283 (33.4%)	103 (28.0%)	180 (37.6%)	0.003
Cardioembolic	408 (48.2%)	214 (58.2%)	194 (40.5%)	< 0.001
Other defined	156 (18.4%)	51 (13.9%)	105 (21.9%)	0.003
Baseline NIHSS	14 (11,19)	15 (11,20)	14 (10,18)	< 0.001
Onset-to-presentation time (Median, IQR, min)	205 (98,378)	152 (73,251)	258 (124,520)	< 0.001
Within 3 h (*n*, %)	376 (44.4%)	213 (57.9%)	163 (34%)	< 0.001
Within 4.5 h (*n*, %)	527 (62.2%)	280 (76.1%)	247 (51.6%)	< 0.001
Within 6 h (*n*, %)	617 (72.8%)	310 (84.2%)	307 (64.1%)	< 0.001
Image modality				
CTA or MRA	560 (66.1%)	129 (35.1%)	431 (90.0%)	< 0.001
CTP or MRP	313 (37.0%)	9 (2.4%)	304 (63.5%)	< 0.001
ASPECTS (median, IQR)	8 (7–10)	9 (8–10)	8 (7–10)	0.071
Intravenous thrombolysis (*n*, %)	306 (36.1%)	185 (50.3%)	121 (25.3%)	< 0.001
Occlusion site (*n*, %)				
ICA	326 (38.5%)	153 (41.6%)	173 (36.1%)	0.106
MCA	496 (58.6%)	211 (57.3%)	285 (59.5%)	0.527
ACA	25 (2.9%)	4 (1.1%)	21 (4.4%)	0.005
Tandem occlusion (*n*, %)	117 (13.8%)	49 (13.3%)	68 (14.2%)	0.713
DPT (median, IQR, min)	101 (75,151)	112 (78,161)	95 (73,141)	<0.001

PSCs indicated primary stroke centers; CSCs, comprehensive stroke centers; SD, standard deviation; TOAST, Trial of Org 10,172 in Acute Stroke Treatment Large Artery Atherosclerosis; LAA, large-artery Atherosclerosis Study; NIHSS, National Institutes of Health Stroke Scale; IQR, interquartile range; CTA, computed tomography angiography; MRA, magnetic resonance angiography; CTP, computerized tomography perfusion; MRP, magnetic resonance perfusion; ASPECT, Alberta Stroke Program Early CT score; ICA, intracranial carotid artery; MCA, middle cerebral artery; ACA, anterior cerebral artery; DPT, door-to-puncture time.

**Table 2:** Endovascular procedure details of the included patients, stratified by type of stroke center.

Procedure	All (*n* = 847)	PSCs (*n* = 368)	CSCs (*n* = 479)	*p* value
First line strategy (*n*, %)				
Stent	677 (79.9%)	316 (85.9%)	361 (75.4%)	<0.001
Aspiration	143 (16.9%)	52 (14.1%)	91 (19.0%)	0.061
Angioplasty	23 (2.7%)	0 (0%)	23 (4.8%)	<0.001
Intra-arterial thrombolysis	4 (0.5%)	0 (0%)	4 (0.8%)	0.137
Successful reperfusion (*n*, %)	726 (85.7%)	282 (76.6%)	444 (92.7%)	<0.001
PRT (mean ± SD)	52 (37,76)	54 (38, 75)	51 (36, 76)	0.585
Procedure complication (*n*, %)	59 (7.0%)	29 (7.9%)	30 (6.3%)	0.359

PSCs indicated primary stroke centers; CSCs, comprehensive stroke centers; PRT, puncture-to-reperfusion time; SD indicates standard deviation.

In terms of AIS workflow, the median OPT was 205 min, and 36.1% (306/847) received IVT. Among the 62.2% (527/847) of patients who presented within 4.5 h, about 58.1% (306/527) received IVT. The median door-to-puncture time (DPT) was 101 min. Overall, 85.7% (726/847) of patients achieved successful reperfusion, 7.0% (59/847) experienced procedure complications, 8.6% (73/847) had sICH, 46.3% (392/847) achieved good clinical outcomes, and 18.9% (160/847) died.

The proportion of patients eligible for EVT based on different selection criteria, including occlusion site, stroke severity (NIHSS and ASPECT), eligibility for intravenous thrombolysis, and OPT was showed Supplementary Table 2. Approximately 34.0% of the 847 patients from our registry meet the following strictest selection criteria: ICA and MCA M1 occlusion, NIHSS scores and ASPECTS ≥ 6, and OPT was within 6 h combined with intravenous thrombolysis.

### Comparisons between the PSCs and CSCs

#### Baseline characteristics

**Figure 2:**
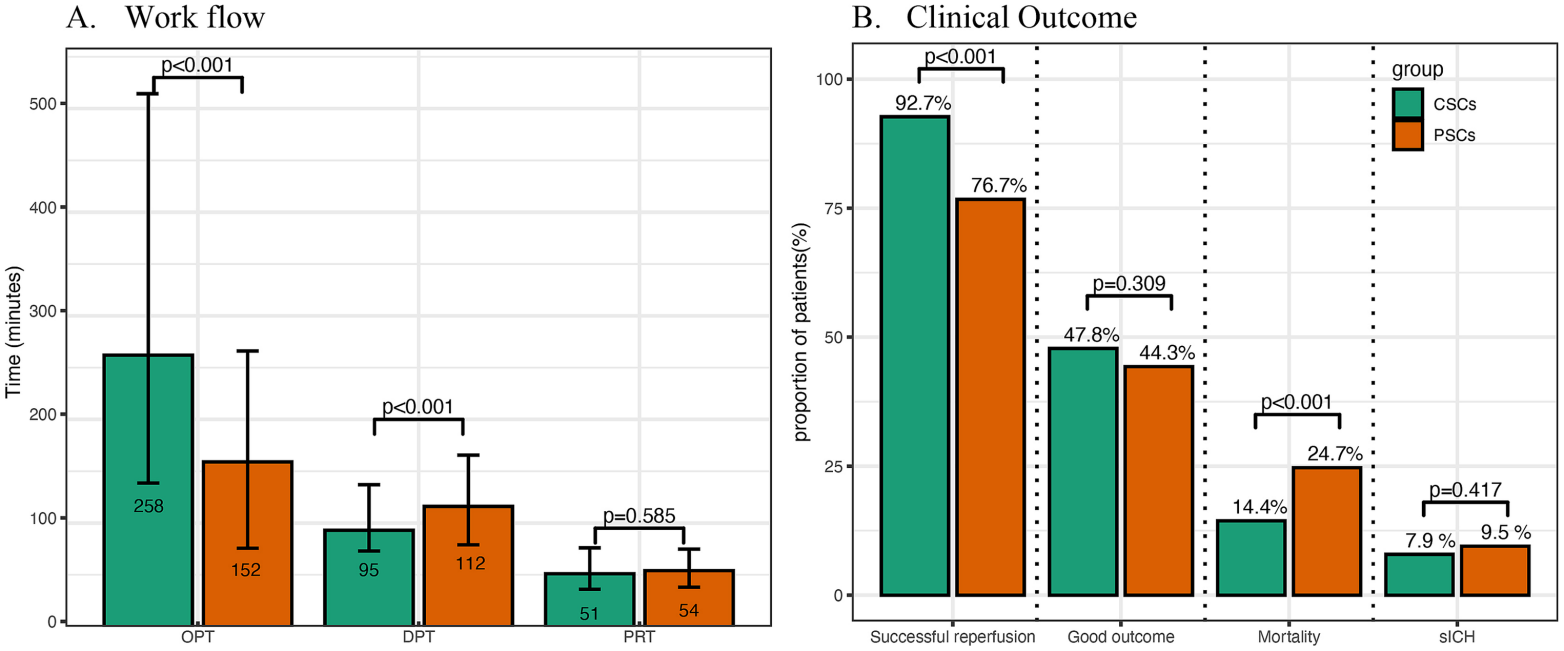
Comparison of workflow, radiological and clinical outcomes between primary and comprehensive stroke centers. OPT indicated onset-to-presentation time; DPT, door-to-puncture time; PRT, puncture-to-reperfusion time; CSCs, comprehensive stroke centers; PSCs, primary stroke centers; sICH, symptomatic intracranial hemorrhage.

Compared with patients from CSCs, those from PSCs had higher prevalence of AF (41.0% vs. 29.9%, *p* < 0.001) and smoking history (33.2% vs. 25.7%, *p* = 0.017). More patients from PSCs presented within 4.5 h (76.1% vs. 51.6%, *p* < 0.001) and 6 h (84.2% vs. 64.1%, *p* < 0.001) of symptom onset. Additionally, a higher proportion of patients from PSCs received IVT (50.3% vs. 25.3%, p < 0.001) and had CE subtype stroke (58.2% vs. 40.5%, *p* < 0.001). Conversely, fewer patients from PSCs had LAA (28.0% vs. 37.6%, *p* = 0.003) or other undefined subtype (13.9% vs. 21.9%, *p* = 0.003). Fewer patients from PSCs underwent CTA/MRA (35.1% vs. 90.0%, *p* < 0.001) or CTP/MRP (2.4% vs. 63.5%, *p* < 0.001). Baseline NIHSS scores were higher in PSCs (median 15 vs. 14, *p* < 0.001), while OPT was shorter (152 min vs. 258 min, *p* < 0.001; Table 1; Figure 2A). However, the DPT was longer (112 min vs. 95 min, *p* < 0.001) in PSCs (Figure 2A).

#### EVT details (Table 2; Supplementary Table 3)

Compared with patients from CSCs, those from PSCs more frequently received stent retrievers (85.9% vs. 75.4%, *p* < 0.001) and less frequently underwent angioplasty as a first-line strategy (0% vs. 4.8%, *p* < 0.001). Successful reperfusion rates were lower in PSCs (76.6% vs. 92.7%, *p* < 0.001; Figure 2B). However, puncture-to-reperfusion time (PRT) (*p* = 0.585; Figure 2A) and procedure complication rates (*p* = 0.359) were similar between the two groups.

#### Clinical outcomes (Tables 3, 4)

**Table 3:** Clinical outcomes of the included patients, stratified by type of stroke center.

Outcomes	All (*n* = 847)	PSCs (*n* = 368)	CSCs (*n* = 479)	*p* value
sICH (*n*, %)	73 (8.6%)	35 (9.5%)	38 (7.9%)	0.417
Good clinical outcome (*n*, %)	392 (46.3%)	163 (44.3%)	229 (47.8%)	0.309
Mortality	160 (18.9%)	91 (24.7%)	69 (14.4%)	<0.001

PSCs indicated primary stroke centers; CSCs, comprehensive stroke centers; sICH indicates symptomatic intracranial cerebral hemorrhage.

**Table 4:** Multivariate logistic regression analyses relationship between clinical outcome and stroke center type.

Parameter	Unadjusted OR (95% CI)	*p*	Model 1 OR (95% CI)	*p*	Model 2 OR (95% CI)	*p*
mRS 0–2	0.87 (0.66–1.14)	0.309	1.00 (0.74–1.35)	1.017	1.04 (0.75–1.43)	0.832
Mortality	1.95 (1.39–2.77)	<0.001	1.83 (1.24–2.70)	0.002	1.76 (1.17–2.66)	0.007
sICH	1.22 (0.75–1.97)	0.418	1.05 (0.63–1.77)	0.842	1.03 (0.60–1.76)	0.929

mRS indicated modified Rankin Scale; sICH, symptomatic intracranial hemorrhage. CSCs as reference. Model 1:adjusted for age, NIHSS, ASPECT；Model 2:adjusted for age, NIHSS, ASPECT, occlusion site, and OPT.

**Figure 3:**
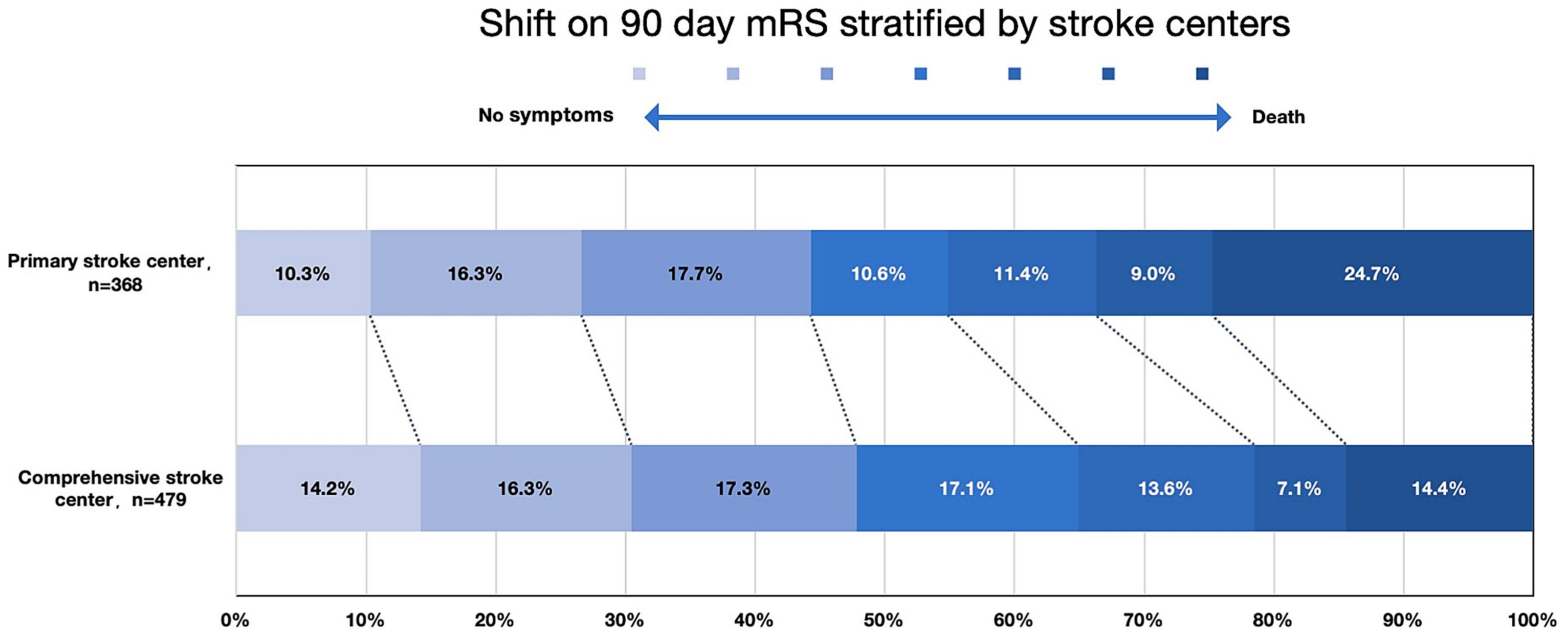
mRS score distribution at 90 days, stratified by stroke center mRS indicated modified Rankin Scale.

The occurrence of symptomatic intracerebral hemorrhage (sICH) (*p* = 0.417) and the rate of good clinical outcomes (*p* = 0.309) were similar between CSCs and PSCs (Figure 2B). The distribution of 90-day modified Rankin Scale (mRS) scores across different types of stroke centers is shown in Figure 3. However, mortality rates were higher in PSCs (24.7% vs. 14.4%, *p* < 0.001). After adjusting for age, NIHSS, ASPECTS, occlusion site, and OPT, clinical outcomes, including good clinical outcomes [adjusted odds ratio (aOR) = 1.04, 95% confidence interval (CI), 0.75–1.43, *p* = 0.832] and sICH (aOR = 1.03, 95% CI, 0.63–1.76, *p* = 0.929), were comparable between CSCs and PSCs. However, the mortality rate remained higher in PSCs than in CSCs (aOR = 1.76, 95% CI, 1.17–2.66, *p* = 0.007; Figure 2B).

### Comparisons of radiological and clinical outcomes between ETERNITY registry and ANGEL-ACT registry

Radiological and clinical outcomes were also compared between ETERNITY registry and ANGEL-ACT registry (Supplementary Table 4). Rate of good outcome and sICH was similar between two registries. Successful reperfusion rate was lowest in PSCs in Eternity registry, highest in CSCs in Eternity registry, general successful reperfusion rate in Eternity registry was comparable with ANGEL-ACT registry.

**Figure 4:**
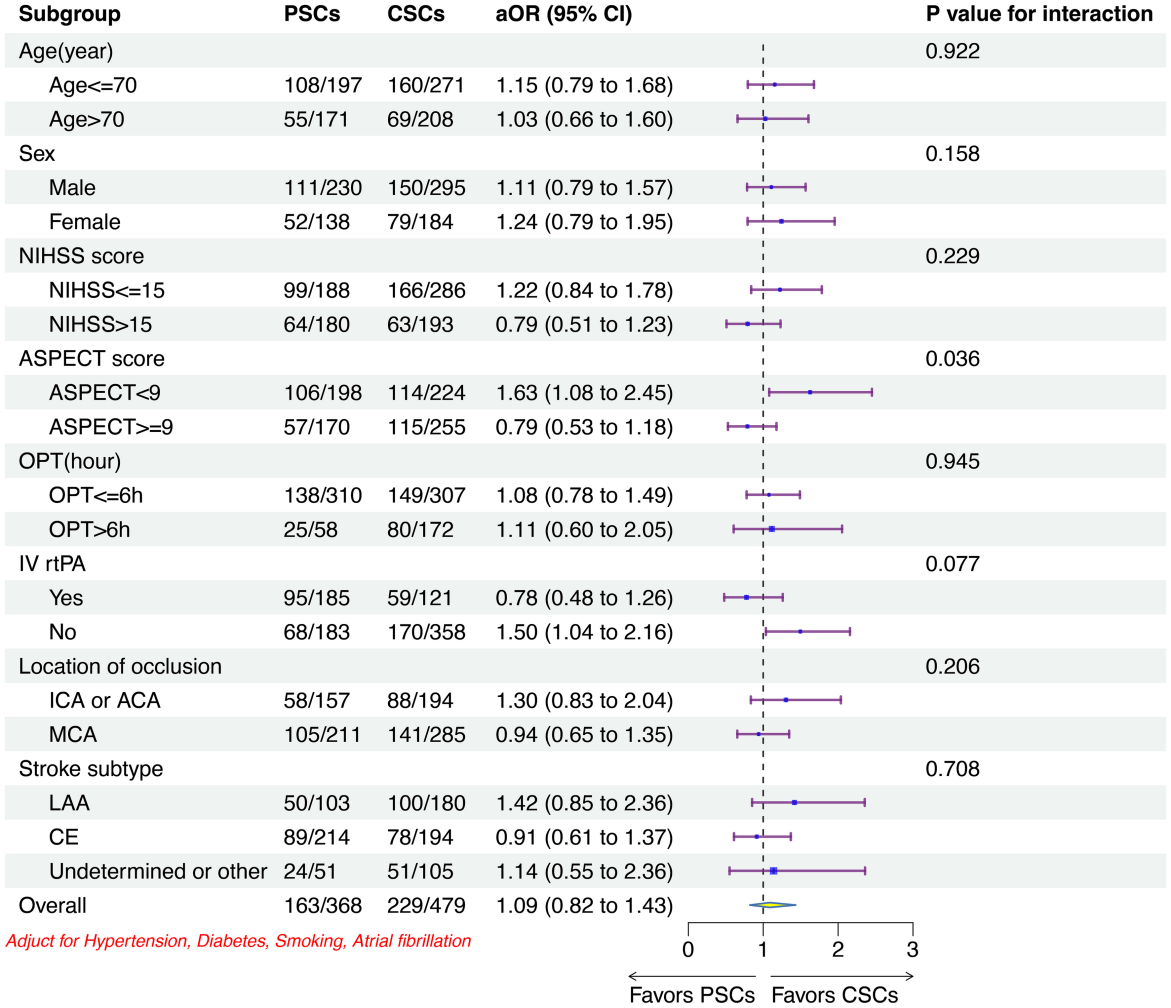
Subgroup analyses of the primary outcome between two stroke centers. CSCs indicated comprehensive stroke centers; PSCs, primary stroke centers; OR, odd ratio; CI, confidence interval; NIHSS, National Institutes of Health Stroke Scale; ASPECT, Alberta Stroke; OPT, onset-to-puncture time; IV, intravenous; rtPA, recombined tissue plasminogen activator; ICA, intracranial carotid artery; ACA, anterior cerebral artery; MCA, middle cerebral artery; LAA, large artery atherosclerosis; CE, cardia embolism.

The clinical outcomes were similar across the predefined subgroups between two type of stroke center, except for patients with ASPECTS < 9 or who no received IVT had more favorable outcomes in CSCs than that in PSCs (Figure 4; Supplementary Figures 1–6).

## Discussion

To our best knowledge, the ETERNITY registry is the first real-world nationwide database with a broad geographic distribution of endovascular capable centers with relative few restrictions for patient selection. It provides an accurate reflection of the current status of EVT in clinical practice both in CSCs and PSCs in China. The registry specifically aims to explore differences in AIS workflow, patients’ characteristic, preprocedural brain image, EVT technique, clinical and radiological outcome between CSCs and PCSs.

From ETERNITY registry, we observed significant differences in patient characteristics between CSCs and PSCs. First, regarding TOAST classification, CE stroke was more common in PSCs, while LAA was more prevalent in CSCs. Second, OPT was shorter in PSCs, with a higher proportion of patients presenting within 4.5 h. Third, rate of IVT, IVT proportion was higher in PSCs. CE strokes typically present with a sudden onset of neurological deficits that are maximal at onset, whereas LAA strokes may have a less defined, waxing and waning course (15). Due to the characteristic onset of CE, patients with CE etiology may be referred to nearby hospitals, which are often PSCs. Combined with better traffic conditions, this may contribute to shorter OPTs and a higher proportion of patients presenting to PSCs within 4.5 h, potentially eligible for IVT.

IVT is a cornerstone treatment for AIS treatment, significantly improving outcomes when administered within 4.5 h of symptom onset (16). However, its utilization in China remains suboptimal. Only 10–20% of AIS patients in China arrive at a hospital within 3 h, and fewer than 3% receive IVT (17). This underuse can be attributed to multiple factors, including delayed symptom recognition, limited awareness among healthcare providers, inefficient in-hospital AIS workflow, and inadequate medical resources. In the ETERNITY registry, approximately 60% of patients arrived at the hospital within 4.5 h, and over 50% received IVT. This higher rate of IVT use in PSCs compared to previous studies (4, 18, 19) likely reflects improvements in stroke care due to the construction of stroke centers in China. The higher proportion of IVT in PSCs may also be influenced by the characteristics of cardioembolic (CE) stroke. CE strokes often present with sudden, severe neurological deficits, prompting quicker hospital presentation. Additionally, better traffic conditions in urban areas where PSCs are often located facilitate faster hospital arrival. The underuse of IVT in eligible population may also be influenced by the perception that direct mechanical thrombectomy (MT) without prior IVT is non-inferior to bridging therapy, as suggested by the DIRECT MT trial, although with a broad non-inferiority margin (20). Moreover, the IRIS meta-analysis showed no superiority of thrombolysis in later time windows (after 2.5 h), further complicating the decision-making process for IVT administration. In summary, while IVT is a highly effective treatment for AIS, its use in China is still limited. The ETERNITY registry highlights improvements in IVT utilization in PSCs, likely due to enhanced stroke center infrastructure and the characteristics of CE stroke. However, continued efforts are needed to optimize IVT delivery and improve stroke outcomes nationwide.

Data from the registry also highlight difference in AIS workflow between CSCs and PSCs. First, regarding brain imaging modalities, angiography and perfusion imaging were less frequently performed in PSCs. Second, DPT was longer in PSCs. Stroke imaging is crucial in AIS management (21). The role of brain imaging in AIS management includes identifying the infarct core (IC), tissue at risk, and arterial lesion (21). Non-contrast CT is an easily accessible and time-saving tool used in AIS management and can be performed at any time. However, it is suboptimal for identifying large vessel occlusions (LVOs) and is less sensitive in detecting extremely early ischemic changes (22). Clinician using NIHSS score ≥ 10 to identify LVO only have 48% sensitivity, potentially missing half of LVO cases (23). Therefore, invasive arterial imaging, such as CTA and MRA, is necessary in AIS management. CTA is the most accurate and efficient non-invasive method for confirming or excluding LVOs and is recommended to be performed as quickly as possible in patients with suspected LVOs (AHA Class I, Level of Evidence B) (23). Perfusion imaging is another important modality for detecting the IC and tissue at risk (24). CTA images analyzed on a PACS workstation with MIP and MPR capabilities may miss about 20% of LVOs (mostly M2 segment) during initial CTA evaluation (25, 26). Perfusion imaging with automated processing software can assist clinicians in easily identifying lesions (27). Since a complete imaging package with non-contrast CT, CTA, and CTP can be obtained in less than 6 min, published endovascular stroke intervention trials have shown that performing CTA imaging does not delay IV tPA infusion (22). Therefore, one-stop multimodal CTA and CTP should be recommended to be performed as soon as possible for better AIS management. However, less than half of AIS-LVO patients in PSCs underwent CTA/CTP. The reasons may include limited awareness among staff in PSCs, medical resource shortages, and in-hospital workflow inefficiencies. Despite the potential time-saving benefits from no advanced brain imaging, DPT was longer in PSCs. The reasons may include the lack of an unobstructed AIS in-hospital workflow, shortage of neuro-interventionists, and absence of a standardized workflow in the catheterization suite. Therefore, the AIS in-hospital workflow, including the emergency department, radiology department, and catheterization suite in PSCs, should be optimized according to the criteria proposed by the CSPPC to minimize door-to-CT, door-to-needle, and DPT time (6). Additionally, training of qualified personnel should be emphasized.

According to the registry, the successful reperfusion rate was lower in PSCs. Our registry showed that more than half of the cases were classified as CE according to the TOAST criteria. Previous studies had demonstrated that compared with no LAA, lower intraprocedural re-occlusion, higher successful reperfusion, shorter PRT (28) in CE stroke. However, data from registry did not show these promising results, on the contrary, low successful reperfusion rate was observed in PSCs. Many factors are associated with successful reperfusion, including occlusion site characteristics; EVT device use, including the use of a stent retriever or balloon guiding catheter; aspiration catheter use; EVT procedural technique (29–37); and neuro-interventionist skill. Effort should be made to improve neuro-interventionists’ capacity by training, enhance EVT success rate via virtual supervision by remote streaming support (38) or utilize remote robotic EVT technology (39). Additionally, it is crucial to ensure that a sufficient range of EVT devices is available to allow the use of appropriate devices according to lesion characteristics.

Despite the longer DPT and lower successful reperfusion rate in the PSCs, the rate of good functional outcomes was similar between PSCs and CSCs. Good functional outcome is associated with multiple factors, including the reperfusion status (13, 40), the onset-to-reperfusion time (41), procedural complications such as vessel perforation (42), and distal embolism (43). In PSCs, the short OPT may mitigate the impact of the lower successful reperfusion rate on overall clinical outcomes.

We also found that nearly one-quarter of thrombectomy cases died in PSCs, with mortality being higher in PSCs. The high mortality rate in PSCs may be attributed to the several factors: first, the high incidence of CE, which is associated with more severe complications and a higher incidence of adverse outcomes (44). Second, the low successful reperfusion rate, which is associated with lager infarct area. Third, post-procedure management. The post-procedure management of AIS, especially in patients with large infarcts is complex and includes blood pressure control, antiplatelet agent administration, brain edema control, and sICH management (42). Fourth, lack of stroke unit. Stroke unit care were associated with 15% lower odds of complications and 15% lower odds of mortality during hospitalization among patients who received thrombolytic therapy (6). Accordingly, to narrow the mortality gap between PSCs and CSCs, we must implement uniform, evidence-based post-reperfusion management protocols and guarantee equitable, round-the-clock access to dedicated stroke-unit beds.

### Limitations

This study had several limitations. First, the registry includes data from only 26PSCs and does not represent all PSCs nationwide. However, we recruited hospitals from 7 regions in China and considered the population density and incidence of AIS morbidity in the different zones, which could provide a representative sample of PSCs nationwide to the greatest extent. Second, we included only AIS patients with anterior circulation LVO who met the 2018 AHA EVT guideline criteria. Hence, the EVT outcomes beyond the recommendations of the guidelines in the real world remain uncertain. Third, the registry included data from only 1 year. As AIS workflow and EVT skills improve annually, additional county hospitals should be recruited, and longer study times are needed to explore the differences between the two types of stroke centers.

## Conclusion

Our nationwide real-world registry highlights several differences in EVT for AIS-LVO patients between PSCs and CSCs in China. Given that the clinical outcomes of AIS-LVO are closely associated with timely intervention and successful EVT, enhancing EVT skills through targeted training programs and standardizing stroke center construction are essential. With further improvements in in-hospital workflow and physician training at PSCs, we anticipate that clinical outcomes for AIS-LVO patients treated at PSCs will improve in the future.

## Data Availability

The raw data supporting the conclusions of this article will be made available by the authors, without undue reservation.
