# Supplementary material for: Comparison of endovascular therapy care and outcome in primary and comprehensive stroke centers for acute ischemic stroke in China a real-world nationwide registry
Source: Front Neurol. 2025 Nov 10;16:1655954. doi: 10.3389/fneur.2025.1655954 (PMC12641255; doi:10.3389/fneur.2025.1655954)
Supplement: Supplementary file 1 [file Table_1.DOCX]

**Endovascular Treatment Differences for Large Vessel Occlusion in Chinese Primary and Comprehensive Stroke Centers: A Real-World Nationwide Registry**

Running Title: EVT differences in two types of Chinese stroke

Table I. Participating centers and enrolled patients

| **Participating centers** | **Province** | **Stroke center type** | **Local PI** | **N=847** |
| --- | --- | --- | --- | --- |
| Zhangzhou Affiliated Hospital of Fujian Medical University | Fujian | Comprehensive stroke center | Wenhuo Chen | 125(14.8%) |
| Jiamusi Central Hospital | Heilongjiang | Comprehensive stroke center | Lihua Xu | 76(9.0%) |
| Songyuan Jilin Oilfield Hospital | Jilin | Comprehensive stroke center | Dongsheng Ju | 56(6.6%) |
| Liaoning Province Health Industry Group Fukuang General Hospital | Liaoning | Comprehensive stroke center | Lin Gao | 50(5.9%) |
| ORDOS Central Hospital | Inner Mongolia | Comprehensive stroke center | Yingchun Wu | 40(4.7%) |
| Jingzhou Hospital Affiliated to Yangtze University | Hubei | Comprehensive stroke center | Junfeng Su | 40(4.7%) |
| Bayinggolin Mongolian Autonomous Prefecture People's Hospital | Xinjiang | Comprehensive stroke center | Xinshan Wu | 28(3.3%) |
| Gansu Provincial Hospital of TCM | Gansu | Comprehensive stroke center | Ya Shao | 26(3.1%) |
| The Tenth Affiliated Hospital of Southern Medical University (Dongguan People's Hospital) | Guangdong | Comprehensive stroke center | Runxiong Li | 19(2.2%) |
| Handan Central Hospital | Hebei | Comprehensive stroke center | Wei Tian | 17(2.0%) |
| Bin Zhou Central Hospital | Shandong | Comprehensive stroke center | Shicong Zhou | 2(0.2%) |
| Yongkang First People's Hospital | Zhejiang | Primary stroke center | Hanming Tu | 52(6.1%) |
| Taihe County People's Hospital | Anhui | Primary stroke center | Shuguang Zhao | 35(4.1%) |
| Longchuan County People's Hospital | Guangdong | Primary stroke center | Wei Linping | 27(3.2%) |
| Xingguo People's Hospital | Jiangxi | Primary stroke center | Guangui Yang | 26(3.1%) |
| Taoyuan Country People's Hospital of Hunan province | Hunan | Primary stroke center | Tanhui | 26(3.1%) |
| Fuqing Hospital Affilited of Fujian Medical University | Fujian | Primary stroke center | Jian Ye | 24(2.8%) |
| Sinan County People's Hospital of Guizhou province | Guizhou | Primary stroke center | Changjun Yang | 21(2.5%) |
| Tangshanfengrun county people's hospital | Hebei | Primary stroke center | Shijun Zhao | 19(2.2%) |
| Xinhui People's Hospital | Guangdong | Primary stroke center | Weidong Huang | 19(2.2%) |
| The Second Hospital of Zhangzhou | Fujian | Primary stroke center | Shujuan Gan | 19(2.2%) |
| Xiu Yan People's Hospital | Liaoning | Primary stroke center | Zhang said hui | 13(1.5%) |
| Tieling County Central Hospital | Liaoning | Primary stroke center | Zhangyi | 10(1.2%) |
| Wudi People's Hospital | Shandong | Primary stroke center | Zhipeng Xu | 10(1.2%) |
| Huazhou People's Hospital | Guangdong | Primary stroke center | Zhiliang Li | 9(1.1%) |
| Longhua County Hospital | Hebei | Primary stroke center | Hailong Wang | 9(1.1%) |
| Xiushui County First People's Hospital | Jiangxi | Primary stroke center | Xiongwei Lu | 8(0.9%) |
| Banan Hospital of Chongqing Medical University | Chongqing | Primary stroke center | Jie Wang | 7(0.8%) |
| Guangshan County People's Hospital | Henan | Primary stroke center | Lei Zhang | 7(0.8%) |
| Jiangyin People's Hospital | Jiangsu | Primary stroke center | Xinmin Zhou | 6(0.7%) |
| Lianzhou People's Hospital | Guangdong | Primary stroke center | Jiyun Feng | 6(0.7%) |
| The Second People's Hospital of Pingnan | Guangxi | Primary stroke center | Yanguang Zhuo | 5(0.6%) |
| Shen zhou People's Hospital | Hebei | Primary stroke center | Zhangbingjun | 3(0.4%) |
| Foshan Sanshui District People's Hospital | Guangdong | Primary stroke center | Wenjun Liang | 2(0.2%) |
| Gongyi People's Hospital | Henan | Primary stroke center | Haiyan Cao | 2(0.2%) |
| Lin Zhou People's Hospital | Henan | Primary stroke center | Guangqiang Song | 2(0.2%) |
| Ningcheng County Central Hospital in Chifeng City | Inner Mongolia | Primary stroke center | Qingsong Zhao | 1(0.1%) |

Table II. Eligibility for Endovascular Therapy

| Criteria | n/N (%) of eligible patients | n/N (%) of eligible patients in PSCs | n/N (%) of eligible patients in CSCs |
| --- | --- | --- | --- |
| -M1 and ICA occlusion  -NIHSS and ASPECTS≥6  -Arrival within 0-6 h  -With intravenous thrombolysis | 288/847(34.0%) | 179/368(48.6%) | 109/479(22.7%) |
| -M1 and ICA occlusion  -NIHSS and ASPECTS≥6  -Arrival within 0-6 h  -With/without Intravenous thrombolysis | 585/847(69.1%) | 302/368(82.1%) | 283/479(59.1%) |
| -M1 and ICA occlusion  -NIHSS and ASPECTS≥6  -Arrival within 0-24 h  -With/without intravenous thrombolysis | 763/847(90.1%) | 358/368(97.3%) | 405/479(84.6%) |
| -All patients with Anterior  Circulation LVO  -NIHSS and ASPECTS≥6  -Arrival within 0-24 h  -With/without intravenous thrombolysis | 783/847(92.4%) | 362/368(98.4%) | 427/479(89.1%) |

NIHSS indicated National Institutes of Health Stroke Scale; ASPECTS, Alberta Stroke Program Early CT Score; mTICI, modified Thrombolysis in Cerebral Infarction Score; mRS, modified Rankin Scale; ICH, intracranial hemorrhage

Table III. Treatment details

| Treatment details | Total | Primary stroke center | Comprehensive stroke center |
| --- | --- | --- | --- |
| Intravenous thrombolysis  Treatment/Arrival within 4.5 h/All | 306/527/847  （58.1%） | 185/247/368  （74.9%） | 121/280/479  （43.2%） |
| Intravenous thrombolysis treatment within 180 min | 249(29.4%) | 157(42.7%) | 92(19.2%) |
| Onset to intravenous thrombolysis | 156.5(106-212) | 169(120-224) | 143(105-205) |
| Endovascular treatment  Stent retriever first  Aspiration first  Angioplasty or stent first  Intra-arterial thrombolysis first | 677(79.9%)  143(16.8%)  23(2.7%)  4(0.5%) | 316(85.9%)  52(14.1%)  0(0%)  0(0%) | 361(75.4%)  91(19.0%)  23(4.8%)  4(0.8%) |

Table IV. Comparison of clinical and radiological outcomes between ETERNITY and ANGEL-ACT registry population with Anterior Circulation LVO

|  | ETERNITY population | PSCs in ETERNITY | CSCs in ETERNITY | ANGEL-ACT population |
| --- | --- | --- | --- | --- |
| mRS 0-2 | 46.3%(392/847) | 44.3%(163/368) | 47.8%(229/479) | 45.4%(607/1337) |
| Successful reperfusion | 85.7%(726/847) | 76.6%(282/368) | 92.7%(444/479) | 87.9%(1227/1396) |
| sICH | 8.6%(73/847) | 9.5%(35/368) | 7.9%(38/479) | 8.9%(118/1325) |
| Mortality | 18.9%(160/847) | 24.7%(91/368) | 14.4%(69/479) | 14.4%(193/1337) |

NIHSS, National Institutes of Health Stroke Scale; mRS, modified Rankin Scale; sICH, symptomatic intracranial hemorrhage.

Figure I. Distribution of 90 days’ mRS at stratified by sex and stroke center type


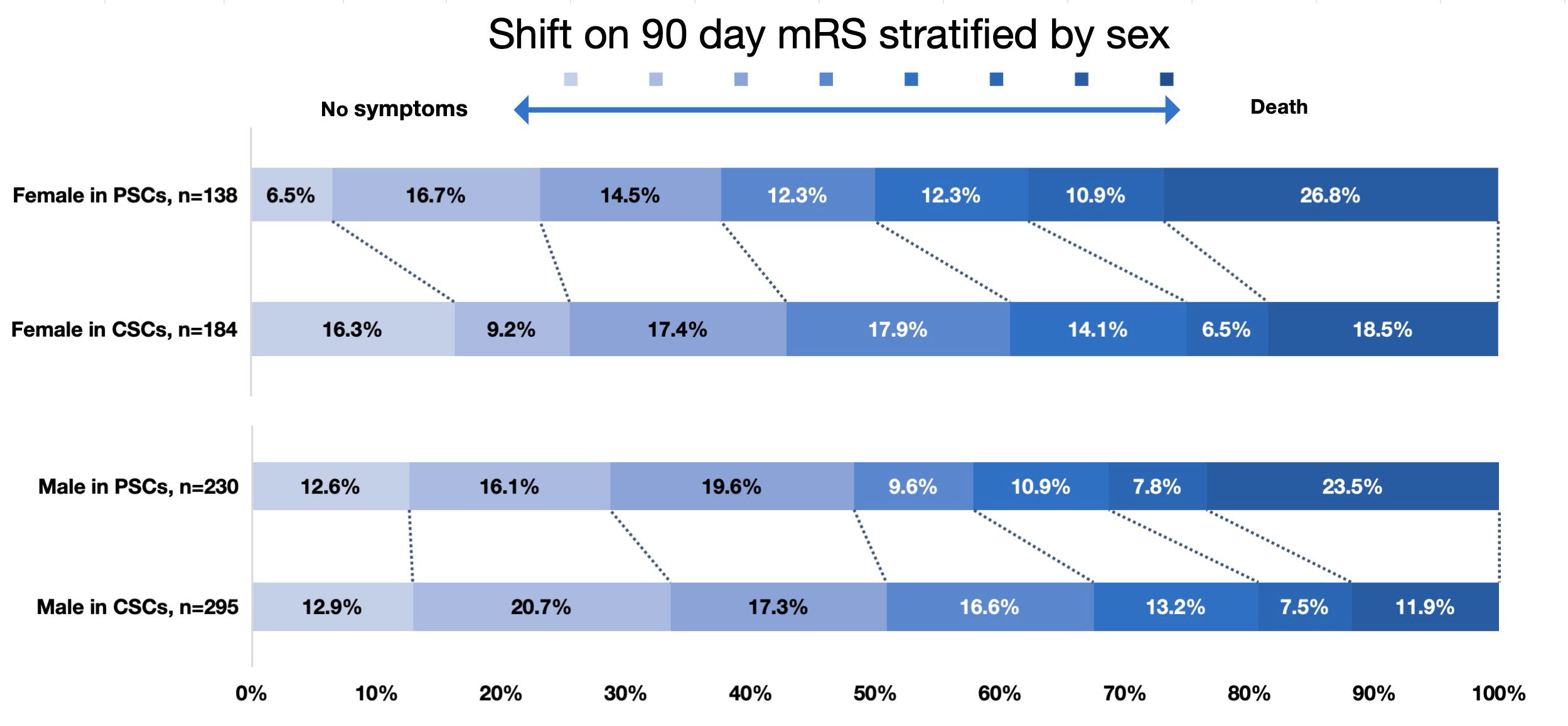


mRS indicated modified Rankin Scale; PSCs, primary stroke centers; CSCs, comprehensive stroke centers.

Figure II. Distribution of 90 days’ mRS at stratified by age and stroke center type


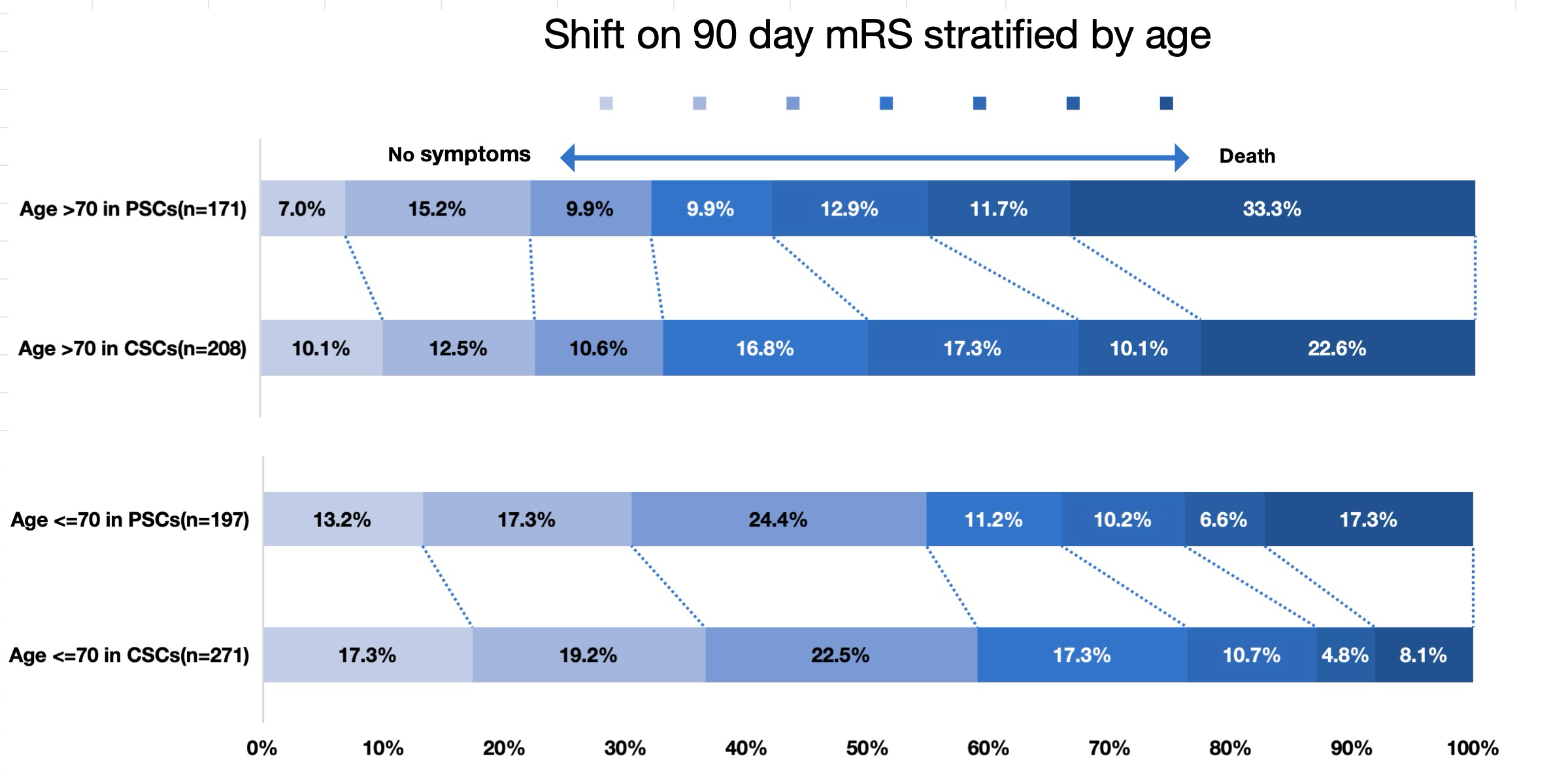


mRS indicated modified Rankin Scale; PSCs, primary stroke centers; CSCs, comprehensive stroke centers.

Figure III. Distribution of 90 days’ mRS at stratified by NIHSS and stroke center type


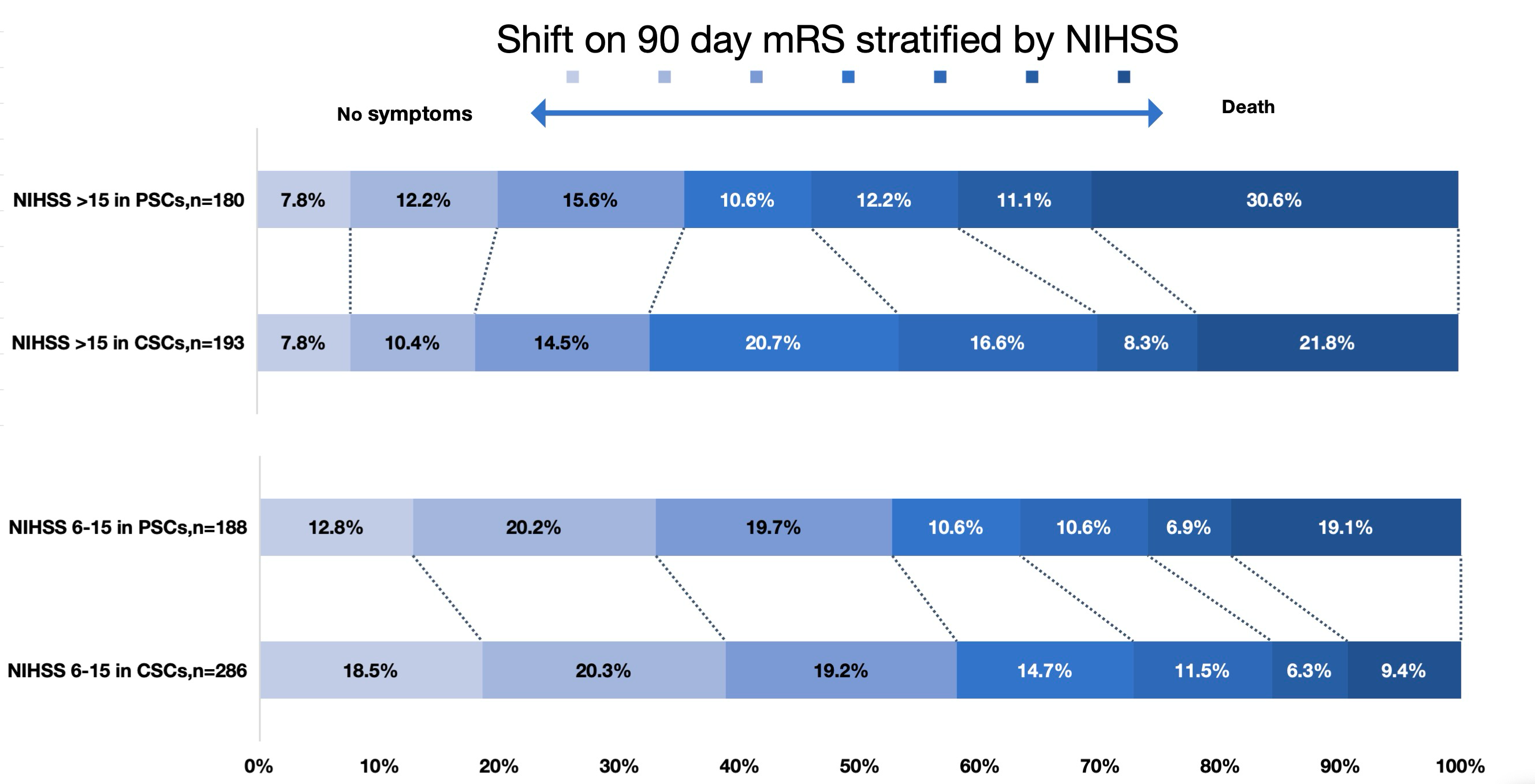


mRS indicated modified Rankin Scale; NIHSS, National Institutes of Health Stroke Scale;PSCs, primary stroke centers; CSCs, comprehensive stroke centers.

Figure IV. Distribution of 90 days’ mRS at stratified by ASPECT and stroke center type


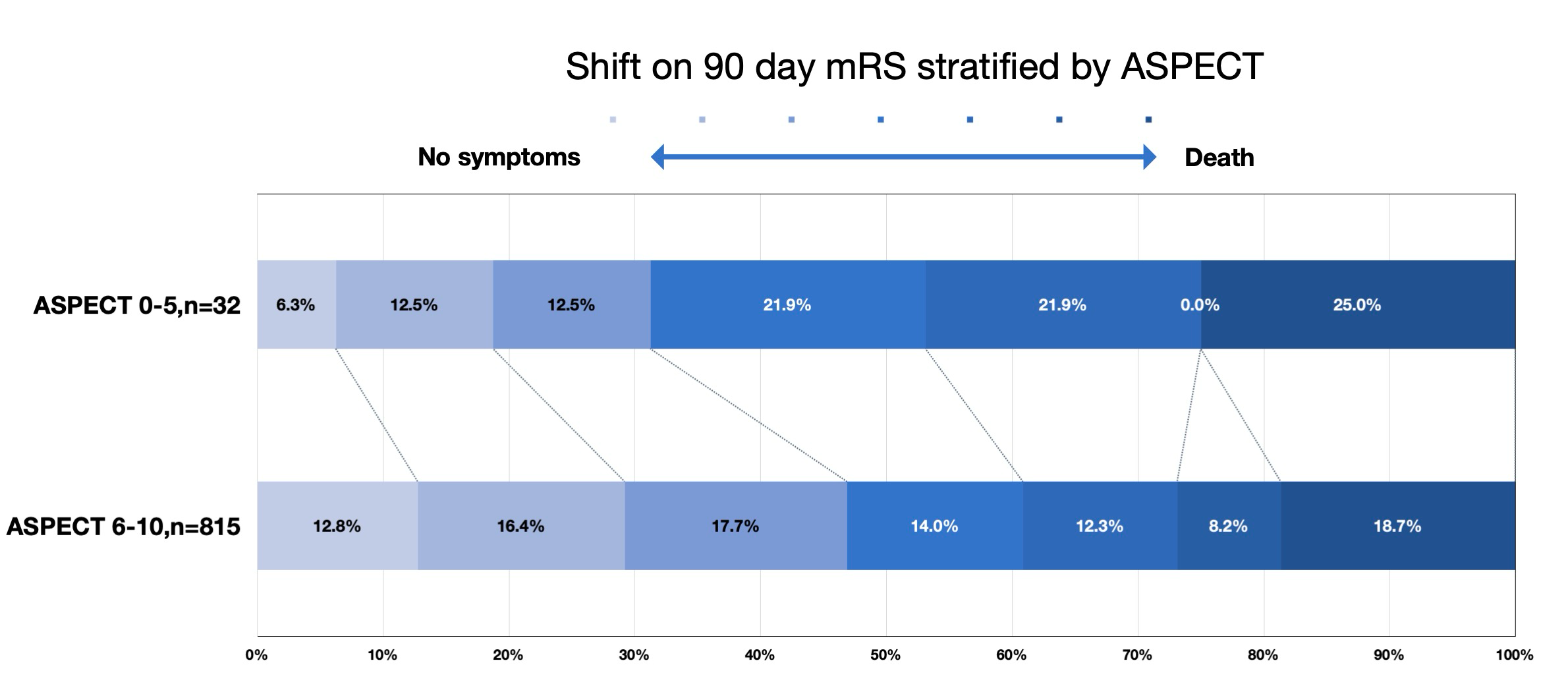


mRS indicated modified Rankin Scale; ASPECT, Alberta Stroke Program Early CT Score.

Figure V. Distribution of 90 days’ mRS at stratified by IVT and stroke center type


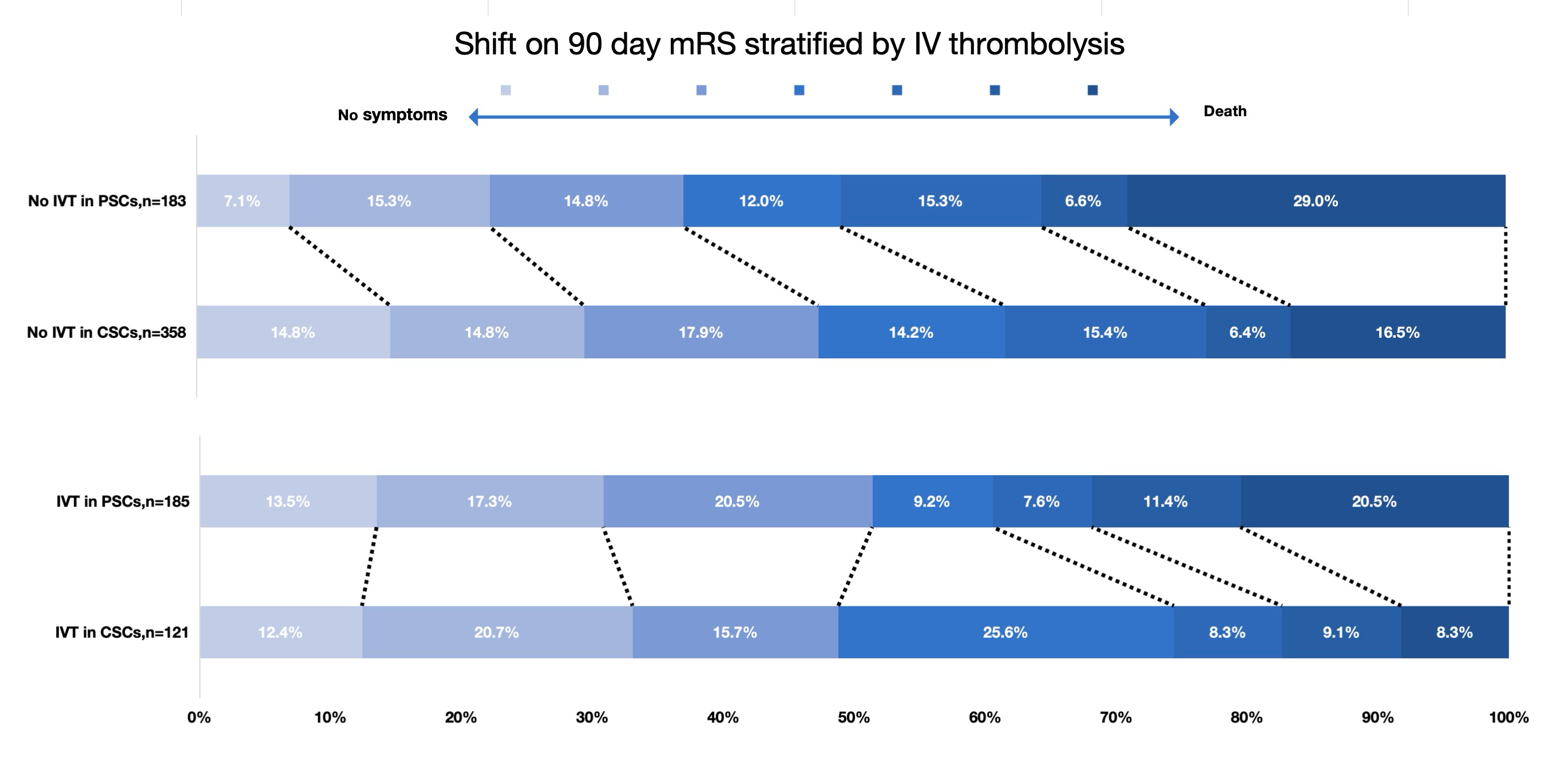


mRS indicated modified Rankin Scale; PSCs, primary stroke centers; CSCs, comprehensive stroke centers.

Figure VI. Distribution of 90 days’ mRS at stratified by onset-to-presentation time and stroke center type


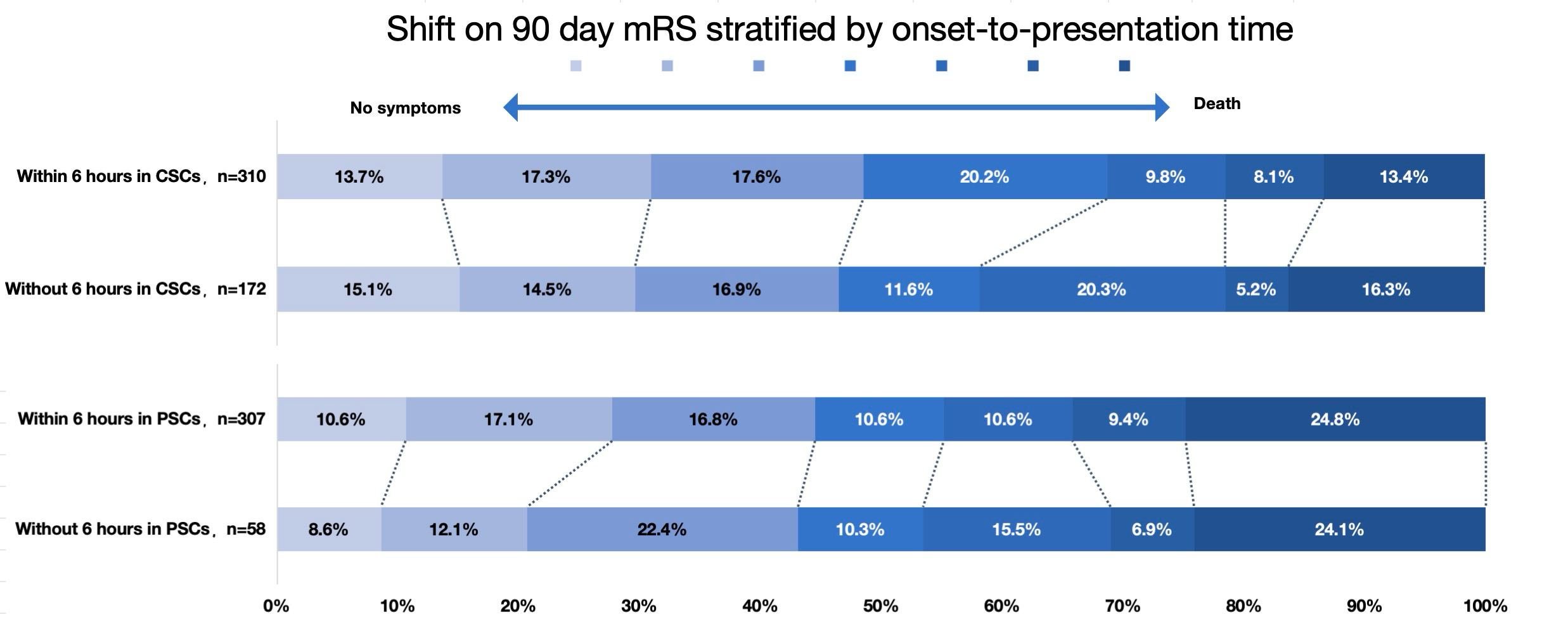


mRS indicated modified Rankin Scale;PSCs, primary stroke centers; CSCs, comprehensive stroke centers.
